# Integrase deficient lentiviral vector: prospects for safe clinical applications

**DOI:** 10.7717/peerj.13704

**Published:** 2022-08-12

**Authors:** Chee-Hong Takahiro Yew, Narmatha Gurumoorthy, Fazlina Nordin, Gee Jun Tye, Wan Safwani Wan Kamarul Zaman, Jun Jie Tan, Min Hwei Ng

**Affiliations:** 1 Centre for Tissue Engineering and Regenerative Medicine (CTERM), Universiti Kebangsaan Malaysia Medical Centre (UKMMC), Kuala Lumpur, Malaysia; 2 Institute for Research in Molecular Medicine (INFORMM), Universiti Sains Malaysia, Pulau Pinang, Malaysia; 3 Department of Biomedical Engineering, Faculty of Engineering, University Malaya (UM), Kuala Lumpur, Malaysia; 4 Advanced Medical and Dental Institute, Universiti Sains Malaysia (USM), Bertam, Kepala Batas, Pulau Pinang, Malaysia

**Keywords:** Integrase-deficient lentiviral vector, Gene therapy, Immunization, Cell Reprogramming, Cell death

## Abstract

HIV-1 derived lentiviral vector is an efficient transporter for delivering desired genetic materials into the targeted cells among many viral vectors. Genetic material transduced by lentiviral vector is integrated into the cell genome to introduce new functions, repair defective cell metabolism, and stimulate certain cell functions. Various measures have been administered in different generations of lentiviral vector systems to reduce the vector’s replicating capabilities. Despite numerous demonstrations of an excellent safety profile of integrative lentiviral vectors, the precautionary approach has prompted the development of integrase-deficient versions of these vectors. The generation of integrase-deficient lentiviral vectors by abrogating integrase activity in lentiviral vector systems reduces the rate of transgenes integration into host genomes. With this feature, the integrase-deficient lentiviral vector is advantageous for therapeutic implementation and widens its clinical applications. This short review delineates the biology of HIV-1-erived lentiviral vector, generation of integrase-deficient lentiviral vector, recent studies involving integrase-deficient lentiviral vectors, limitations, and prospects for neoteric clinical use.

## Introduction

Lentiviral vector (LV) or integrase-proficient lentiviral virus (IPLV) is a viral vector commonly derived from human immunodeficiency virus-1 (HIV-1) (Liu & Berkhout, 2014). The HIV-derived LV is first reported for its functions in *in vivo* gene delivery and stable transduction of non-dividing cells (Naldini et al., 1996). The vector is packaged by transfection of packaging elements that code for viral particles like HIV. While IPLV processes genomic integration of the vector DNA *via* the integrase enzyme, integrase-deficient lentiviral vector (IDLV) is dedicated to the reduction of genomic integration. IDLV is formed from a disabled, reduced efficiency integrase enzyme and vector RNA (Apolonia, 2020). As a consequence of reduced genomic integration, the resulting vector DNA remains in the nucleus of the transduced cell and does not get replicated during cell proliferation (Dong & Kantor, 2021). Together with the removal of non-essential viral elements in the packaging vector and introduction of self-inactivating LTR (SIN-LTR), these “inert” characters of the vector not only provide safety and stability but also support transient expression of protein in the transduced cell. These properties allow IDLV to be exploited and improvised for application in clinical studies and therapeutics, such as gene delivery and gene therapy. Thus, there is a need to introduce and summarize the studies on the characteristics of IDLV and its current application. We intend to produce a narrative review to enable scientists and medical officers to explore the possibility of IDLV in clinical applications. In this short narrative review, we will describe the general biology of lentiviral vectors and existing derivatives of IDLV systems, as well as elaborating the applications of IDLV in the recent studies for possible clinical implementation.

## Survey/Search Methodology

**Figure 1:**
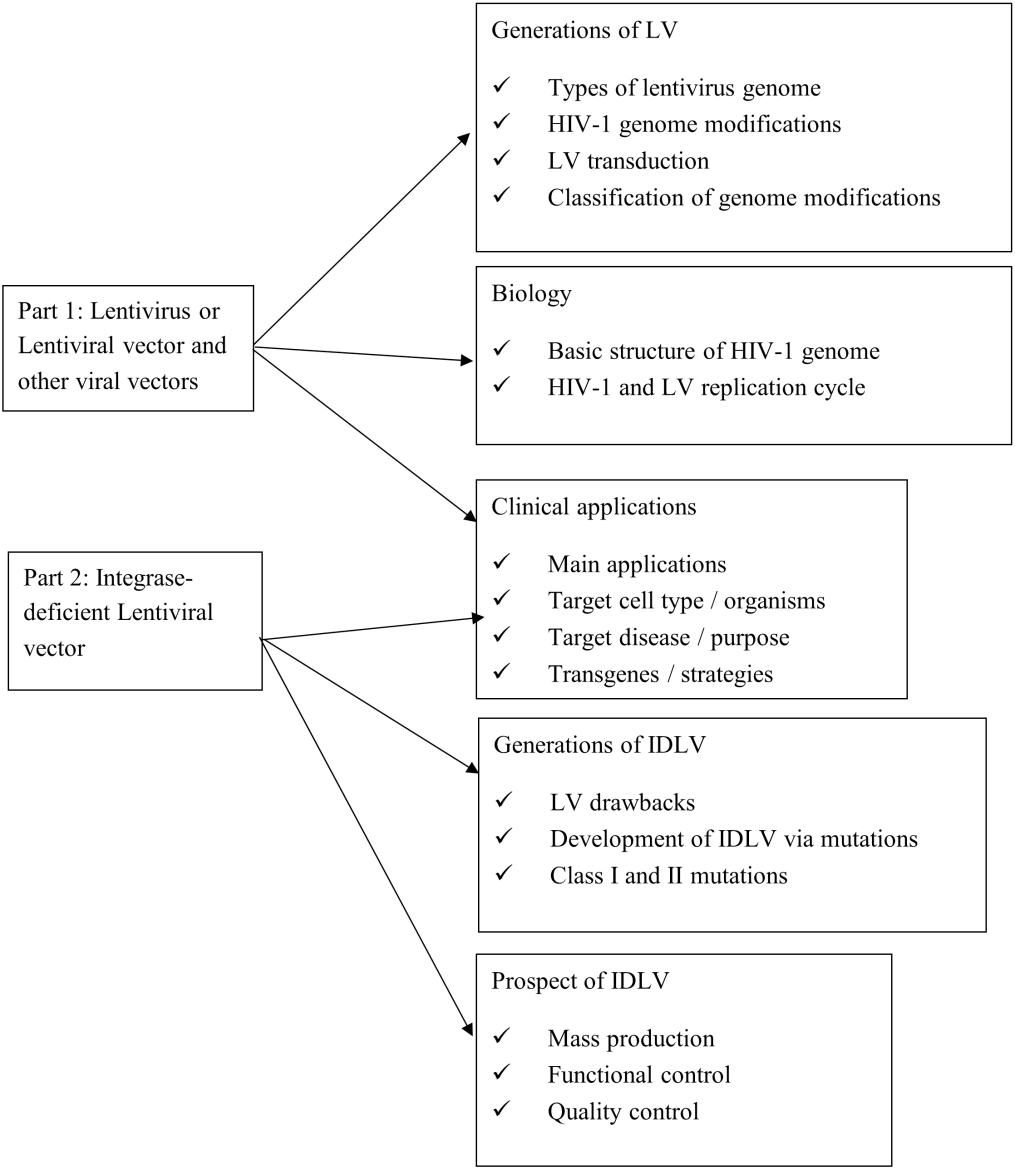
Survey/search methodology.

In this narrative review, we searched the relevant studies in the following electronic databases: Google Scholar, US National Library of Medicine (PubMed) as well as the website clinicaltrials.gov on current and past trial studies. Due to the limited studies for IDLV applications, we searched through the database with studies published in the latest 15 years. When referring to the origin and fundamental studies, we did not limit the published year. As depicted in Fig. 1, we divided our review into two parts where the first part (‘Human Immunodeficiency Virus 1 and Lentiviral Vector’) is composed from analysis of search about lentivirus or lentiviral vector. We started the search by looking up lentivirus and lentiviral vectors as our search phrase. We then collected the relevant studies that cover the biology, generations, and clinical application of lentiviral vectors. Subsequently, the second part (‘Production of IDLV’) focuses on searching with IDLV as the main keyword. We further collected, assembled, and categorized the IDLV studies based on the biology of IDLV production and showing feasibility on clinical application (‘Applications of IDLV for Clinical Implementations’). While there is little current real world clinical application of IDLV, we further search for studies that support the prospect of IDLV as a potential candidate in clinical trials. The search terms are related to the mass production with quality or functional control and regulations as a probable drug (‘Limitations and Prospects’).

 For the analysis of data, we retrieved the studies by skimming through the abstract to look up the main application of study, the targeted cell type or organism, the targeted disease or purpose of study and the transgenes or strategy used to tackle the targeted disease or experimental purpose. We further extracted and tabulated the information pertaining to the above in the article’s main body.

This study has been conducted with the support and approval from Universiti Kebangsaan Malaysia (UKM) (Ethic approval code UKM PPI/111/8/JEP-2021-347).

## Human immunodeficiency virus 1 and lentiviral vector

**Figure 2:**
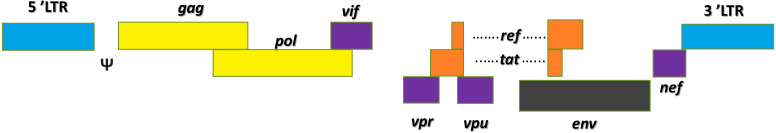
HIV-1 genome contains nine genes. The genes of HIV are in the central region of the pro-viral DNA. These proteins are divided into three classes: the major structural proteins (Gag, Pol, and Env); the regulatory proteins (Tat and Rev) and the accessory proteins (*Vpu, Vpr, Vif,* and *Nef*).

Lentivirus is a genus of retrovirus. It is an enveloped virus with two copies of single-stranded RNA genome, which upon infection is reverse transcribed into cDNA in the host cell’s cytoplasm (Stoye et al., 2011). The infamous HIV-1 belongs to the lentivirus genus (German Advisory Committee Blood, 2016). Figure 2 shows the basic structures of the HIV-1 genome. Its viral genome consists of nine open reading frames that span across the structural and enzymatic genes, *gag, pol*, and *env*, that are common to all retroviruses. The *gag* gene encodes three main structural proteins: matrix (MA), capsid (CA), and nucleocapsid (NC) while the pol gene encodes the protease (PR), reverse transcriptase (RT), and integrase (IN). These enzymes play critical roles in the cleavage of viral polypeptides, virus maturation, reverse transcription of viral RNAs and the integration of provirus into the cellular genome (Katz & Skalka, 1994). The *env* gene encodes the structural proteins which are the SU (surface) and TM (transmembrane) protein, and it is responsible for the virus attachment to its host, allowing virus entry to the cell (Park, 2007; Park & Goto, 2006). Two other genes, *tat* and *rev,* which encode for two regulatory proteins, complete transcriptional function and efficient nuclear export of unspliced or singly spliced messenger RNA (mRNA) (Annoni et al., 2007). Four accessory genes: *vif, nef, vpr,* and *vpu* play vital roles in the immunogenicity and replication of the virus (Tomás et al., 2013). Each end of the RNA genome of HIV-1 contains 5′ and 3′ long terminal repeats (LTRs), with an untranslated 5′ and 3′ region (U5/(U3) flanking repeat elements (R). The packaging signal ‘*ψ*’ is located between the 5′-LTR and the *gag* gene (Sakuma, Barry & Ikeda, 2012). Transcribed mRNAs are processed by cellular machinery and exported out of the nucleus with the help of Rev responsive elements (RRE) as a *cis* element and finally the viral mRNAs get synthesized. The viral genome and proteins are assembled at the plasma membrane and release new viral particles from the host cell (Sakuma, Barry & Ikeda, 2012). The multimerization of *gag* and *gag-pol* proteins activates the viral PR shortly after virus budding, leading to structural rearrangements and form mature infectious virions.

Vectors based on viruses are promising vehicles for gene transfer applications due to their specific cell tropism and efficiency in gene delivery applications. The HIV-1 genome has been widely used to produce lentiviral vectors. LV derived from HIV-1 is constituted by several non-coding sequences that control gene expression and protein synthesis (Zufferey et al., 1998). The HIV-1 genome was modified by removing some components or adding new components to generate different generations of LV with safety features and enhanced functions. One goal of biosafety measures is avoiding the emergence of replication-competent lentiviruses. To decrease the formation of replication competent lentiviruses and its adverse consequences, deletion of some accessory proteins and splitting of the viral genome into multiple plasmids were first applied. Modifications to the original lentivirus biology has classified the vector virus into four different generations with possible functions and benefits as tabulated in Table 1. The first two generations consist of three plasmids, the third generation consists of four plasmids and the fourth generation has five separate plasmids. There are few existing modifications to improve the current four generation systems, such as *rev*-independent *gag-pol* expression related codon optimization, involvement of Simian immunodeficiency virus (SIV) accessory protein *vpx*, and different packaging elements for altered vector tropism (Sakuma, Barry & Ikeda, 2012). Additionally, the central polypurine tract (cPPT) or the central termination sequence (CTS) within the *pol* sequence contribute to the efficient reverse transcription, central DNA flap production, increased vector transduction efficiency and infection of non-dividing cells (Follenzi et al., 2000; Liu & Berkhout, 2014; Zennou et al., 2000). The enhancer/promoter sequence such as cytomegalovirus (CMV) within the U3 region of the 5′ LTR allows gene expression (Tomás et al., 2013). A self-inactivating (SIN) vector harbours a deletion in the U3 region of the 3′ LTR that contains the TATA box and binding sites for transcription factors Sp1 and NF- *κ*B (Hamid, Kim & Shin, 2017). During reverse transcription, this deletion is transferred to the 5′ U3 sequence producing provirus without LTR region (Zufferey et al., 1998). Thus, this deletion will prevent the 5′LTR from transcribing full-length viral genomic RNA, which will eventually disrupts viral replication. To compensate for the loss of *tat*, a chimeric 5′LTR was replaced in the U3 region with a strong heterologous internal promoter such as cytomegalovirus (CMV) or Rous sarcoma virus (RSV) as these promoters directs high-level expression in a broad range of tissues and cells (Dull et al., 1998).

**Table 1:** Elements in lentiviral vector systems of four different generations.

**Generations**	**Plasmid system**	**Elements added and its Function**	**Elements removed and its importance**
First	3 plasmids system	Expression cassette LTR sequence from HIV-1 genome, encapsulation sequence, RRE sequence, and a promoter to drive transgene expression.	
				Envelope plasmid Replaced HIV-1 envelope with VSV-G (vesicular stomatitis virus glycoprotein) envelope to transduce a wide range of cell types.	
		Packaging plasmid Sequences of regulatory proteins (*tat, rev*), structural proteins (*gag, pol*), and accessory proteins (*vif, vpr, vpu, nef*)	
Second	3 plasmids system		Packaging plasmid Removed all accessory proteins (*vif, vpr, vpu, nef*) that are non-essential proteins involving in viral replication
Third	4 plasmids system	Addition of HIV-1 cPPt and CTS *pol* gene -Important for the central DNA flap production -Enable viral DNA nuclear import and infection of non-dividing cells. -Rev protein is provided by a different plasmid. -increased vector transduction efficiency	Packaging plasmid -removed all accessory proteins (*vif, vpr, vpu, nef*) and 1 regulatory gene (*tat*). -reduce insertional mutagenesis -reduce RCL formation
		Woodchuck hepatitis virus post-transcriptional regulatory element (WPRE) -Enhances mRNA transcript stability. -Increases transgene expression	Expression cassette -inactivation of LTR -substitution of U3 region by CMV/RSV promoters from HIV-1 5′ LTR -reduce insertional mutagenesis -eliminates *tat* dependence
		Replacement of *Rev/RRE* by autonomous RNA export signals (aka constitutive RNA transport elements (CTE)	TATA box, Sp1 and NF-kB transcription factor -from HIV-1 3′ LTR -Known as self-inactivating vectors (SIN)
Fourth	5 plasmid system	*gag-pro* and *pol* are further separated into two plasmids -The *pol* gene is fused to *vpr* to ensure transport of the reverse transcriptase/integrase protein into the recombinant viral particle. -High expression of essential viral components are driven by 2 separate plasmids: the *Tet-Off* and *Tat* transactivators.	-reduce the recombination events that leads to RCL formations -reduce autonomous replication of virus

**Figure 3:**
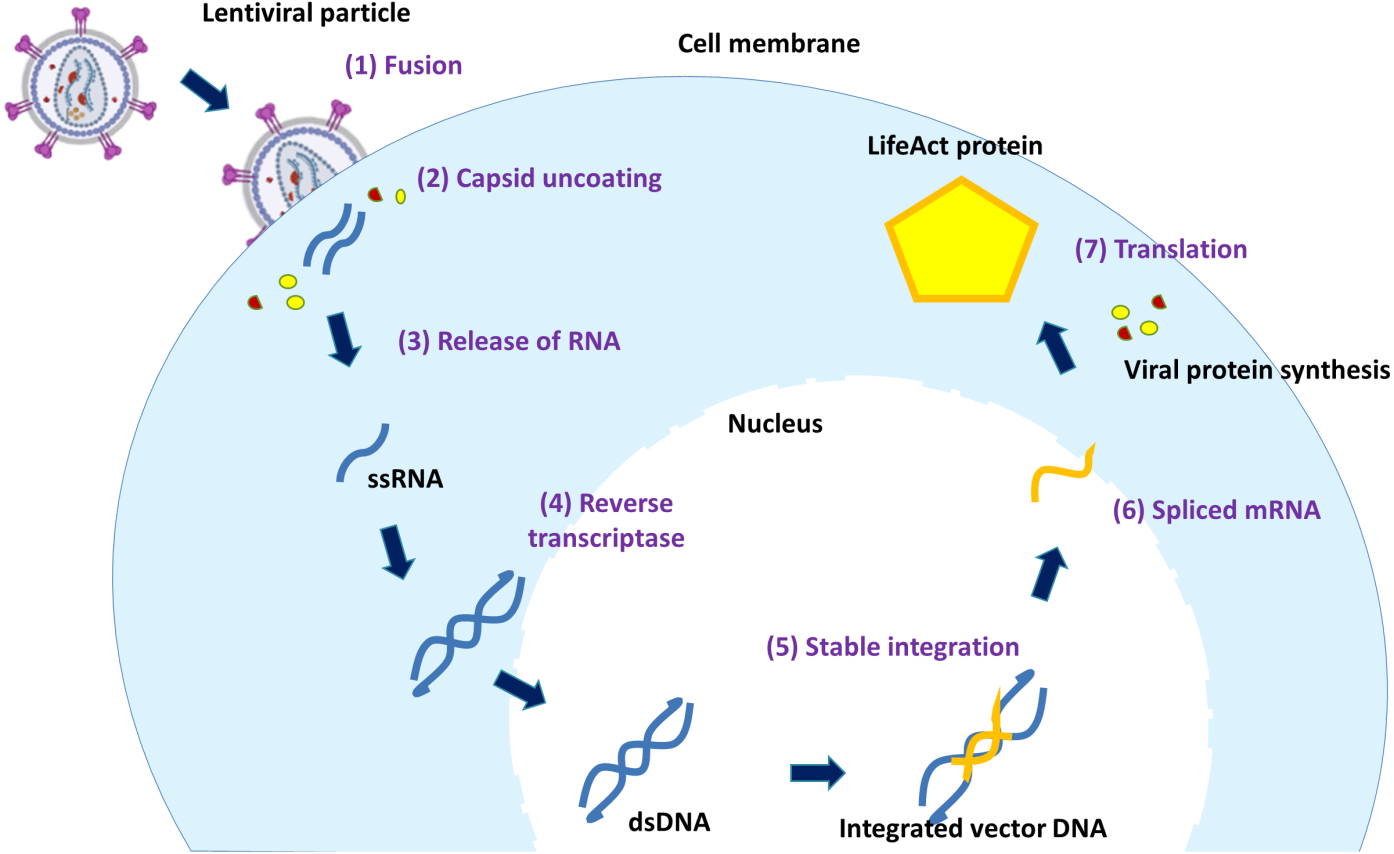
The mechanism of lentiviral transduction. Stages of lentiviral transduction. Schematically shown are seven stages of lentiviral transduction including: (1) viral fusion to a receptor/coreceptor, (2) endocytosis of the vector & uncoating of capsid proteins, (3) release of RNA genome, (4) the positive sense RNA is converted by RT into double-stranded DNA in the cytoplasm, (5) the viral vector entry to the nucleus and & integration into the host genome, (6) fully spliced viral mRNAs can be exported from the nucleus to the cytoplasm, (7) translation.

Figure 3 depicts the transduction process of lentiviral vector. The process starts as the vector virus binds to the host cell outer membrane. Once the vector virus fuses with the host cell membrane, capsid proteins (CA) are first remodelled with some host partners such as Nup153 and CPSF6 at the nuclear compartment to form multimeric CA that aids in nuclear translocation and successful intranuclear journey of the viral particle (Scoca & Di Nunzio, 2021). The uncoated and remodelled particle releases RNA genome and viral enzymes (RT, IN and PR) (Sakuma, Barry & Ikeda, 2012). Vector RNAs are processed by cellular machinery after reverse transcription. They are converted to cDNA by the expressed RT, transported into the nucleus and inserted into the host genome by IN. Once the vector DNA is integrated into the host DNA, the resulting integrated vector DNA divides along with the host cell chromosome.

Throughout the development of four generations of lentiviral vectors, there are more splitting of original viral genes to different strands for safer vector production. However, new LV generations with low concentration yield need thorough research for clinical trials. In addition to the inhibited vector replication, future LV systems may have codon optimization and more splitting of genes for regulating vector production while retaining their transducibility and flexibilities.

## Production of IDLV

LV has efficient and safe clinical trial profile especially in gene therapy. However, LV may also develop unexpected adverse effects, like insertional mutagenesis due to disruption in cellular physiology. Recently, a gene therapy case involving a patient’s hematopoietic stem cells transduced with the BB305 lentiviral vector for sickle cell disease adversely developed Acute myeloid leukaemia (AML). The peripheral-blood samples revealed that the blast cells contained a BB305 lentiviral vector insertion site although the research team believed that leukaemia was unlikely to be related to vector insertion (Philippidis, 2021). Thus, it is vital to avoid insertional mutagenesis from gene dysregulation at the LV integration site within or near a coding region of the host genome (Schlimgen et al., 2016). Additionally, the enhancers at LTR region on the vector DNA, which were added to enhance the transcription activity, may also upregulate the targeted oncogenes (Cavalli & Misteli, 2013). The promoter at 5′ LTR can dysregulate transcription initiation, and form oncogenic chimeric transcripts with host sequences by alternative splicing through the usage of HIV constitutive and cryptic splice sites (Cavazzana-Calvo et al., 2010; Moiani et al., 2012). Yang et al. (2007) found that U3 region deletion in SIN LVs not only decreased transcriptional termination but also increased generation of chimeric transcripts. Preclinical and clinical studies suggested that the insertion of retroviral splicing and polyadenylation signals within transcription units may cause posttranscriptional deregulation of gene expression (Almarza et al., 2011). For example, LV insertion caused posttranscriptional activation of a truncated proto-oncogene in a *β*-thalassemia patient, resulting in benign clonal expansion of hematopoietic progenitors (Cavazzana-Calvo et al., 2010). The chimeric mRNAs in this study were generated *via* cryptic splice sites which disrupt the original host sequence aberrantly and transform transduced cells into cancerous form (Moiani et al., 2012). Although there are unlikelihood of genetic predisposition of LVs, the propensity of LVs to integrate into the transcribed genes increases the probability of insertional mutagenesis events.

Despite numerous demonstrations of good safety profile of integrating lentiviral vectors, precautionary solution has been developed to generate integrase-deficient versions of these vectors. Integration-deficient lentiviral vectors (IDLVs) typically have mutations in the IN gene. Without the functioning IN enzyme, the vector DNA will exist as non-replicating episomes in transduced cells and unable to enter host cell genome. Dividing cells therefore will lose the vector DNA gradually and reduce the risk of any possible insertional mutagenesis (Banasik & McCray, 2009).

**Figure 4:**
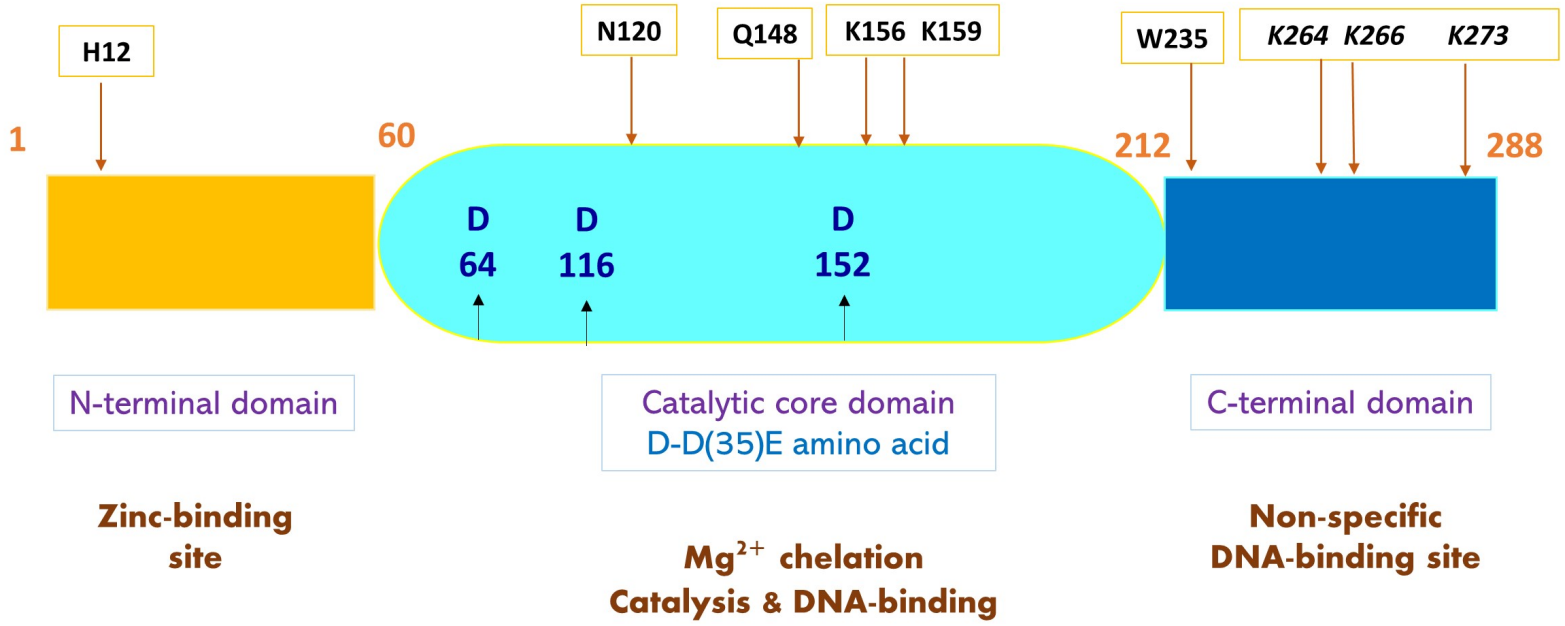
A schematic representation of HIV-1 integrase (IN) and mutated amino acids to produce IDLV. Structure of HIV-1 IN comprises three functional domains: N-terminal domain containing zinc-binding site that binds viral DNA, the catalytic core domain containing the D-D35E amino acids, and C-terminal domain containing DNA-binding site that binds non-specific target DNA-binding sites. Sites of IDLV mutations are indicated by arrows showing amino acids that give rise to mutations. Class I integrase mutations present at catalytic IN-core domain (middle) are in blue bold. Mutations affecting DNA binding and strand transfer are in black and italicized. Mutations at W235 and N120 blocks genomic binding while Q168 mutations impair vector DNA binding. H12 mutation at N-terminal domain affects IN-multimerization.

IDLV can be designed by introducing mutations in the LTR integrase attachment site (Δatt) (Fig. 4). There are many types of point mutations that can be induced to residues in the three domains: the N-terminal domain, the core domain, and the C-terminal domain to produce IDLVs. These mutations can be divided into Class I and Class II mutations. There are three types of class I mutations (also known as the IN-core domain): D64, D116 and E152. Among the three, D64 mutations is most used in gene therapy. Class I mutations introduced to the integrase enzyme negatively impacts the machinery required for recognition, binding and recombination between the provirus, host DNA and integrase (Engelman, 1999). This was done by changing the conserved residues in the N-terminal domain of HIV-1 IN, whereby, this action was able to block the virus replication (Engelman, 2011). The reduced vector integration efficiency has promoted the yield and expression of episomal vector DNA in the form of linear or circular DNA (Banasik & McCray, 2009). Additional mutations affect IN genomic or DNA binding, linear episome processing,S or IN multimerization. IN must successfully bind both vector DNA and genomic DNA to support integration. Mutations at W235, N120, and RRK (262-264) specifically block the genomic DNA binding. The alteration of H12 affects the IN multimerization to generate IDLVs. In addition, K264/K266/K273 mutations (also known as a triad) have been proven to impair both DNA binding and strand transfer. For successful IN-mediated integration to occur, the vector LTRs are cleaved at an invariable CA dinucleotide. Mutations in these LTRs (att mutants) also render the vector integration deficient (Nightingale et al., 2006). Vector DNA binding is also impaired by altering Q148. Class II mutations can be performed by exchanging the standard gag or pol packaging plasmid with an IN-mutant strain (Banasik & McCray, 2009). Several studies reported that mutations in specific amino acids of IN resulted in impaired integration of lentiviruses (Engelman et al., 1995; Goubin & Hill, 1979). Class II mutations can also affect the reverse transcription and impair the vital viral life cycle stages and cause pleiotropic effects that can make them unsuitable for vector developments (Engelman & Craigie, 1992).

Since there are a few numbers of possible mutations, it is important to compare the transducing efficacy and integration rates of IDLVs from different amino acid substitutions. However, there are limited studies that compared these IN mutations because there are also other cofactors that affects the transducing efficacy and integration rates of the IDLVs. The efficiencies vary with the type of promoter, envelopes used for different types of diseases and the target cells for a study. A complete review by Shaw & Cornetta (2014) listed the multiple studies conducted with distinct IDLV modifications thus far. The reason for type of IDLV modification chosen in each study reported was not specifically stated and therefore, it is difficult for us to choose one best modification based on previous reports. However, one study conducted in 2014 reported that D167H IN displayed the most intriguing phenotype with an increased integration rate of about 40% as compared to WT vectors as it was well conserved in HIV. This study found that vector with D64V substitution was two to six times more efficient for cell transduction than vectors Q168A, N, or LQ (Saeed et al., 2014). D64V showed the strongest inhibition of integrase without affecting the vector DNA synthesis. Compared to other mutations, the D64V mutation allows transductions efficiency to only be slightly reduced than the integrating one.

Overall, IDLV is developed as an alternative to vector-mediated integration (Banasik & McCray, 2009). The main advantage of IDLV is to reduce unintended consequences such as dysregulated cell function or potential activation of proto-oncogenes due to random integration within chromosomes. Similar to self-inactivating (SIN) LVs on reducing upregulation of proto-oncogenes, researchers also found the ultimate advantage of IDLV in many pre-clinical and clinical applications (Cousin et al., 2019; Liu & Berkhout, 2014; Negri, Michelini & Cara, 2010; Philippe et al., 2006; Sarkis et al., 2008; Vijayraghavan & Kantor, 2017). Despite the improved safety from potential oncogenesis, reduction of integration also limited the number of cells that succeeded transduction when compared to integrating vectors. Thus, IDLV would be a better option if its limitations are properly studied, and its advantages are considered in future clinical applications.

## Applications of IDLV for clinical implementations

**Figure 5:**
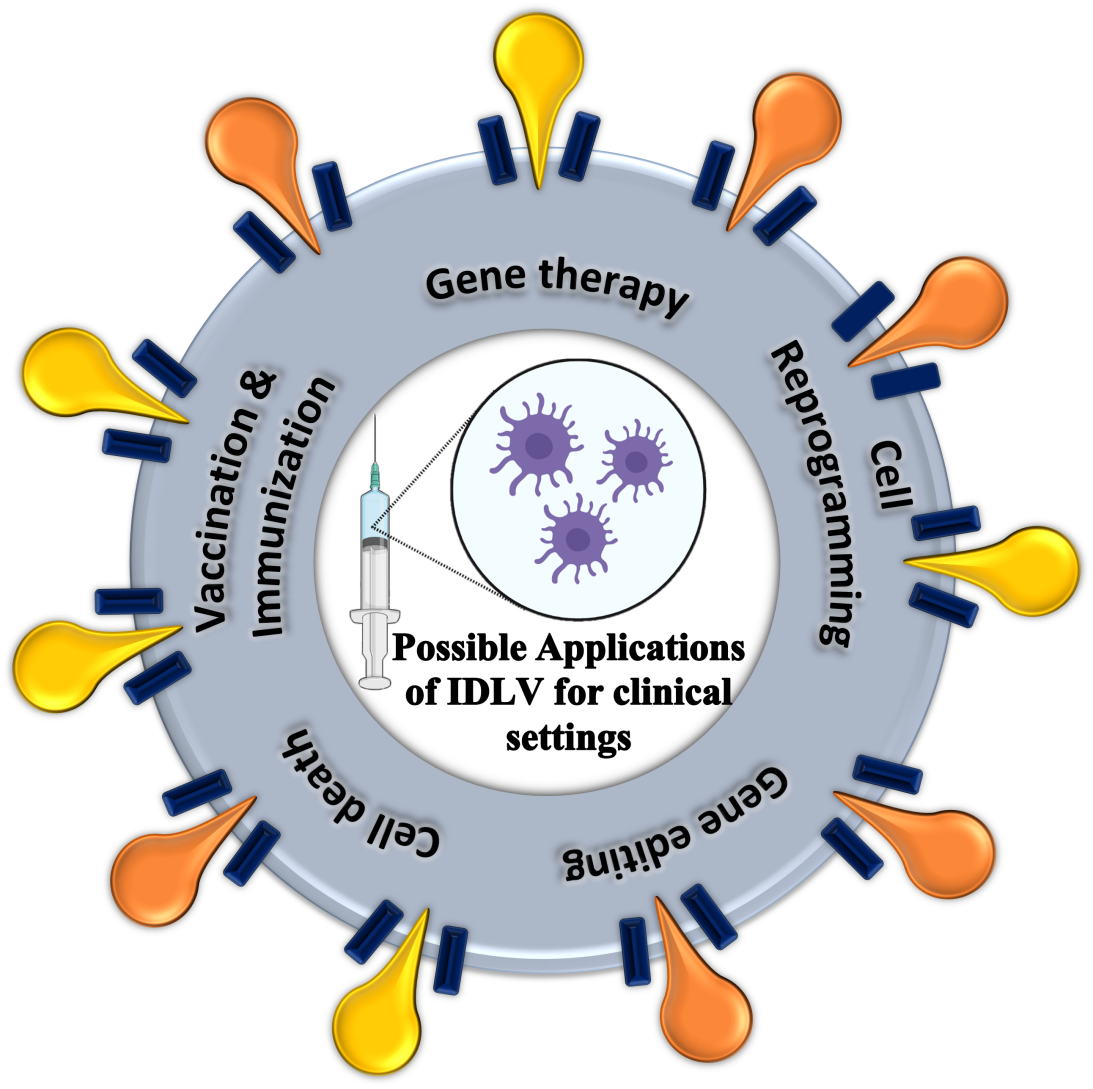
Possible applications of IDLV for clinical settings. The five current applications possible for implementations in clinical settings, include gene therapy, cell reprogramming, gene editing, cell death and vaccination or immunization.

While there is wide use of common viral vectors, such as those derived from adenovirus, IPLV, SIV and some other retrovirus in clinical applications, recent studies also utilize IDLV as a vehicle to deliver transgenes into the transcriptional location of the cells (Table 2). Due to defective IN, these transgenes normally remain in the nucleus and are targeted for transcription and translation without directed access to the genome of the cell. The transcription and translation of these transgenes promoted desired functions in the cells. These functions may serve for the studies of gene editing, gene integration, gene therapy, immunization, or vaccination, promoting cell death, cell reprogramming, *etc.* (Fig. 5).

**Table 2:** Recent studies of IDLV for possible clinical applications.

**Main application**	**Target cell type/organism**	**Targeted disease/purpose**	**Transgenes/strategy**	**Ref.**
Gene therapy *(Transient expression)*	U937 cells	–	*eGFP*	Nordin et al. (2016) and Nordin, Karim & Wahid (2014)
		Retinal Pigment epithelium in mice	Rpe65 deficiency	*eGFP and Rpe65*	Yanez-Munoz et al. (2006)
	Haemophilia B Mice	Factor IX (FIX) deficiency of haemophiliac V	*Human FIX cDNA*	Suwanmanee et al. (2014)
Cell Reprogramming	HepG2 cells, fibroblast	Induce pluripotent states that resembles human embryonic stem (hES) cells	Yamanaka’s factors, SV40 Tag	Mali et al. (2008)
		U937 cells	Induce pluripotency	Yamanaka’s factors	Nordin (2011)
		Bone marrow MSC	Induce pluripotency via Yamanaka’s factors but flanked by loxP sites between LTRs	*Cre* recombinase	Papapetrou & Sadelain (2011)
	Hepatic progenitor cells	Sort and select human hepatic progenitor cells	*GFP* driven by liver-specific apolipoprotein A-II (*APOA-II*) promoter	Yang et al. (2013)
Cell death	293T cells	Selective elimination of tumour cells	Thymidine kinase (*TK*) suicide gene	Vargas Jr, Klotman & Cara (2008)
Gene editing *(Flp recombinase)*	HEK-293 and 293T	Integration of circular DNA	Flp recombinase	Moldt et al. (2008)
Gene editing (*I-SceI nuclease*)	U2-OS, HEK293, HT-1080, and HEK293T cells	Defective enhanced green fluorescent protein (EGFP) gene	I-SceI nuclease and eGFP	Cornu & Cathomen (2007)
Gene editing (*Sleeping Beauty (SB) transposase*)	HeLa cells	Abrogate chromosomal	Sleeping Beauty (SB) transposase	Vink et al. (2009)
Gene editing (*Piggyback (PB) DNA transposase*)	HEK293 and HeLa cells	Puromycin screening	Puromycin resistant gene	Skipper et al. (2018)
Gene editing *(Zinc Finger Nuclease)*	Epstein-Barr virus–transformed B lymphocytes (lymphoblastoid cells), cord blood CD34+ hematopoietic progenitor cells, human ES cell lines (HUES-3 and HUES-1)	Severe combined immunodeficiencies (SCID)	*IL2RG with* a silent point mutation, *GFP* or *PuroR*	Lombardo et al. (2007)
		Human repopulating hematopoietic CD341 stem cells	X-linked severe combined immunodeficiency (SCID-X1)	*AAVS1, IL2RG*	Genovese et al. (2014)
	CD34+ hematopoietic cells	Fanconi anaemia (FA)	*FANCA* expression cassette	Diez et al. (2017)
Gene editing (*Transcription activator-like effector nuclease (TALEN)*)	Patient derived T-lymphocytes	X-linked hyper-immunoglobulin M (hyper-IgM) syndrome (XHIM)	*CD40LG*	Kuo et al. (2018)
	Primary fibroblast	Recessive dystrophic epidermolysis bullosa (RDEB)	*COL7A1*	Osborn et al. (2013)
Gene editing (*Clustered regularly interspaced short palindrome repeats-Cas9 (CRISPR-Cas9))*	Defective keratinocytes	Junctional epidermolysis bullosa (JEB)	*LAMB3*	Benati et al. (2018)
		B-lymphocytes	Eradicating tumour cells	*a-PD1*	Luo et al. (2020)
		HEK293	Off-target nuclease activity sites	Puromycin-resistance gene	Wang et al. (2015)
		HEK293T cells and post-mitotic brain neurons	Off-target nuclease activity sites	All-in-one (sgRNA and *Cas9*) vector cassette with *SP1* binding site	Ortinski et al. (2017)
		Colorectal cancer stem cells (CRCSC)	*in vivo* silencing, investigating the stem-like tumour exosomes secretion	*RAB27B*	Cheng et al. (2019)
		CRC patient-derived xenografts (PDXs)	Gene silencing	*ARID3B*	Liao et al. (2020)
		Induced pluripotent stem cell (iPSC) derived from patients with alpha-synuclein gene (SNCA) locus triplication	Disease model of Parkinson’s	CRISPR-dCas9-DNA methylation system	Tagliafierro, Zamora & Chiba-Falek (2019)
	Sickle cells	Correction of mutation	*βs-globin*	Uchida et al. (2021)
Immunization or vaccination	BALB/c mice	HIV-1 infection	Codon optimized HIV-1JR-FL gp120	Negri et al. (2007)
		Mice	West Nile Virus (WNV) infection	Gene encoding secreted form of envelope of virulent strain of West Nile Virus (WNV)	Coutant et al. (2008)
		Mice	HCV infection	HCV E1/E2 pseudotyped IDLV bearing HCV NS3 gene	Deng et al. (2013)
		CB6F1 mice	Influenza virus	IDLV pseudotyped with virus hemagglutinin (HA) and nucleoprotein (NP)	Gallinaro et al. (2018)
		Mice	Influenza A virus (IAV)	Genes encoding for VN04-2 monoclonal antibodies	Michelini et al. (2020)
		C57BL/6J mice	SARS-COV-2 virus	LV-derived virus simulating particles (VSPs) decorated with SARS-COV-2 spike protein and carrying spike encoding mRNA	Yin et al. (2020)
		Mice and golden hamsters	SARS-COV-2 virus	Spike glycoprotein gene	Ku et al. (2021c)
		Mice	Lymphoma and melanoma	Ovalbumin (*OVA*), *TRP2*	Morante et al. (2021)
	C57BL/6 mice	Anti-tumour immunity	*OVA*	Cousin et al. (2019)

### Gene editing/integration/therapy/screening/trapping

#### Transient expression of desired genes

IDLV has facilitated therapeutic applications *via* the delivery of corrected genes of interest into the desired cells with disease phenotype. As IDLV reduces the integration of the transgene into the genome of the cell, the unintegrated transgene is processed into a stable circular DNA. The circular DNA remains in the cell until the new cell cycle begins, thus the expression of the transgene in circular DNA will yield a long-term phenotype in non-dividing cells. For example, Yanez-Munoz et al. (2006) has successfully delivered and expressed eGFP and Rpe65 *in vivo* in the retinal pigment epithelium *via* IDLV. The efficient expression remains stable from 7 days after transduction and up to 9 months. In contrast to untreated contralateral eyes in mouse with Rpe65 deficiency, electroretinography showed the ERG b-wave response at 100 mcds/m^2^ was almost sixfold greater in vector treated eyes. Noteworthily, a clinical trial to study the safety and efficacy of IDLV based gene therapy for retinal and choroidal neovascularization diseases has been recruiting participants. The trial study employed IDLV to deliver VEGFA antibody gene into RPE cells, which is then expressed to neutralize the VEGFA activity in the posterior segment of the eye of individuals with various forms of neovascular macular degeneration (ClinicalTrials.gov Identifier: NCT05099094).

In another study, Suwanmanee et al. (2014) corrected factor IX (FIX) deficiency in haemophiliac B mice with human FIX expressed by cDNA delivered *via* IDLV without immunity and liver damage implication. The potency of human FIX is enhanced 50-fold by optimizing the cDNA codon usage and generation of highly catalytic mutants. IDLV is also employed in cancer therapeutics, enabling transient expression of therapeutic genes. For example, deoxycytidine kinase (DCK) is introduced to phosphorylate gemcitabine (dFdC) into toxic metabolites that chemosensitize pancreatic adenocarcinoma cancer cells (Hanoun et al., 2016). Another study attempted to deliver transgene encoding diphtheria toxin A, an immunotoxin that induces apoptosis *via* protein translation inhibition together with Survivin promoter that is dramatically promoted in different cancer cells (Lin et al., 2015). Although safety is insured by reduced integration, the expression of transgene in circular form may not be as efficiency as IPLV. Fazlina et al. compared the eGFP expression in U937 cells transduced by SFFV-promoter driven IDLV and IPLV and observed a 50% decrease of fluorescent cells in 10 days post-transduction (Nordin et al., 2016; Nordin, Karim & Wahid, 2014). Thus, specific follow-up amelioration is needed to ensure comparable transgene expression in those by IPLV.

#### Site-mediated gene editing/integration/screening/trapping

Many genetic mutations-driven defective proteins impact the downstream functions in the cells, giving undesirable complications to the affected individual. These mutations can be corrected through gene editing or gene integration to replace the erroneous sequence in the genome of the cell. The editing or integration process involves enzymes that target the flawed sequence, generate double-stranded breaks (DSB) and subsequently activate repair mechanisms of the “broken” genome such as non-homologous end-joining (NHEJ) or homology-directed recombination (HDR) (Danner et al., 2017). A correct or desired genetic piece is then introduced and guided to the site of DNA breakage and participate in the replacement of the original flawed sequence. With the reduced integration rate of transgene, IDLV safely delivers the desired sequence into the specific cell that has its genome “nicked” for mediating repair. Popular gene-editing methods may involve zinc-finger nuclease (ZFN), transcription activator-like effector nuclease (TALENS), clustered regularly interspaced short palindrome repeats (CRISPR)-CRISPR associated protein 9 (CRISPR-cas9) system or transposable elements (Anzalone, Koblan & Liu, 2020; Gupta & Shukla, 2017).

##### Zinc-Finger Nuclease (ZFN).

Zinc-finger nuclease (ZFN) is an artificial restriction enzyme that combines a zinc-finger binding DNA domain and a DNA cleavage domain (Miller et al., 2007; Paschon et al., 2019). The zinc-finger binding domain recognizes the targeted site *via* array fingers (3–4 bps) while the DNA cleavage domain acts to cleave the targeted site. ZFN is generally designed to disable or rewrite alleles. Lombardo et al. (2007) used IL2RG-targeted ZFN to generate cells that expressed inserted corrected genes (delivered by IDLV) *via* HR and showed a good rate of integration (up to 50%) in a panel of human cell lines. Following Lombardo, Gabriel et al. (2011) carried out a genome-wide analysis (GWAS) on ZFN specificity and found off-target at clustered-integration sites bearing homology with the ZFN target sites. Here, the off targets are associated with ZFN and independent of IDLV insertion. Genovese et al. (2014) applied the ZFN gene editing in human repopulating hematopoietic CD34+ stem cells from patients with X-linked severe combined immunodeficiency (SCID-X1) to introduce the therapeutic AAVS1 or IL2RG gene *via* IDLV delivery. Further transplant of the gene-corrected cells into mice showed human cell engraftment, thus showing compatibility in *ex vivo* therapy. Diez et al. (2017) investigated the phenotypic correction from the insertion of FANCA expression cassette in CD34+ hematopoietic cells from Fanconi anaemia (FA) patients *via* two therapeutic IDLV donors. These two IDLV donors contain different genes (eGFP and puromycin) that are used for the screening of edited cells.

##### Transcription activator-like effector nuclease (TALEN).

Like ZFN, transcription activator-like effector nuclease (TALEN) is a fusion nuclease between a transcription activator effector (TALE) binding domain and a DNA cleavage domain (Joung & Sander, 2013). TALE is found in *Xanthomonas* bacterial infection in plants where it recognizes the promoter site of genes that aids the infection. Due to the simple recognition mechanism of TALE with the DNA sequence, it can be engineered to re-target different sites or applied in cells of other plant species, and even human cells. While Kuo et al. (2018) applied IDLV or adeno-associated virus (AAV) and TALEN to integrate CD40LG into the genomes of patient-derived T-lymphocytes, IDLV is also used to capture off-targets of TALENS that is involved in the clustered integration site analysis *via* IDLV trapping. When IDLV delivered a promoter-less reporter gene, which is flanked by a 3′ splice acceptor (SA), into the target site *via* repair machinery upon nuclease induced breakage, expression of the targeted gene is spliced prematurely during the transcription process, leaving expression of a truncated gene (thus trapping) and the reporter gene. Osborn et al. also used TALENS to correct Type VII collagen gene (COL7A1) associated with epidermolysis bullosa. During the investigation, only cells co-introduced with TALENS and IDLV carrying GFP generate a stable GFP expressed population (Osborn et al., 2013).

##### Clustered regularly interspaced short palindrome repeats-Cas9 CRISPR-Cas9).

Recently, the CRISPR-Cas9 system has been practically robust for editing the genome of many organisms (Ebrahimi & Hashemi, 2020; Lino et al., 2018). It was first discovered as an adaptive immune system in bacteria and later applied broadly with other modifications to engineer genomes and regulate the expression of genes (Zhan et al., 2019). The mechanism of CRISPR-Cas9 operates *via* CRISPR targeting RNA (crRNA), trans-activating RNA (tracrRNA) activating and guiding Cas9 protein to the cleaving site, and Cas9 protein cleaving the targeted site subsequently. In the CRISPR type II system, the crRNA and tracrRNA combine to become a single guide RNA (sgRNA) that guides the Cas9 protein to the target site with recognition of a 3 nt protospacer adjacent motif (PAM) site. Cas9 creates DSB on the DNA which is then proceeded with NHEJ or HDR. A modified nuclease deficient Cas9 (dCas9) protein is also used to repress or activate the transcriptional site. IDLV is then used to transduce the donor template to the DSB repair site. Benati et al. restored basement membrane heterotrimeric laminin 332 component, *i.e., LAMB3* gene in the defective keratinocytes *in vitro* with junctional epidermolysis bullosa (JEB) *via* integration of functional gene at the intron site, directed by CRISPR-Cas9 and gene delivery by IDLV (Benati et al., 2018). The grafting of the gene-corrected cells into immunodeficient mice displayed normal dermal-epidermal junction and proves potential *ex vivo* clinical applications. Luo et al. (2020) delivered donor transgenes for *a*-*PD1* antibodies and CRISPR-Cas9 into B-lymphocytes by IDLV and promoted the differentiation to long live plasma cells (LLPCs), which would then show effectiveness in eradicating tumour cells. Wang et al. (2017) showed high HDR frequencies of >80% in human embryonic stem cell (hESC) line WA09 when donor template is transduced *via* IDLV. Here, the efficiency of various mutant deficient IN in IDLV to recruit the cellular protein LEDGF/p75, which mediates the HDR process, is investigated. As the CRISPR-Cas9 system is still limited by its off-target DSB, modification of the system and monitoring is necessary. For this reason, off-target screening is done by integrating screening genes using IDLV delivery *via* DSB generated by the site-directed nuclease system and then followed by mapping of the transgenes in the integrated sites. For instance, Wang et al. (2015) delivered puromycin-resistance gene by IDLV to HEK293 cells to screen the off-target sites generated by unspecific DSB from CRISPR-Cas9 and TALENS systems. While Cas9 and sgRNA are commonly delivered to the target cells separately, Ortinski et al. (2017) performed GFP gene knockout by transducing the whole CRISPR-Cas9 system into HEK293T cells and post-mitotic brain neurons *in vivo* with high efficiency *via* IDLV bearing an all-in-one (sgRNA and Cas9) vector cassette with SP1 binding site. IDLV has yielded short term nuclease activity that is sufficient to induce permanent edit to the targeted DNA, thus considerably safer from generating more off-target DSB compared to integrating vector. Referring to Ortinski, Cheng et al. (2019) performed *in vivo* silencing of RAB27B gene by IDLV-transducing cas9-sgRNA vector cassette into colorectal cancer stem cells (CRCSC) to investigate the stem-like tumour exosomes secretion that promotes tumorigenesis and tumour fitness. Liao et al. (2020) also utilized IDLV-CRISPR/Cas9 system to perform gene silencing of ARID3B in CRC patient-derived xenografts (PDXs). IDLV transduction of a combined system consisting of CRISPR-Cas9 tethered to DNA methyltransferase has also enabled disease model study for Parkinson’s disease. Tagliafierro, Zamora & Chiba-Falek (2019) has differentiated induced pluripotent stem cell (iPSC) derived from patients with alpha-synuclein gene (SNCA) locus triplication into Parkinson’s disease (PD)-relevant dopaminergic neuronal populations by IDLV transducing CRISPR-dCas9-DNA methylation system that epigenetically modulate the regulation of SCNA expression. To correct genes in sickle cell disease, Uchida et al. (2021) designed a cas9-sgRNA-donor RNA system delivery based on IDLV to introduce correction of mutation of *β*-globin gene in sickle cells.

##### Other nucleases or transposase.

Apart from the gene-editing systems, IDLV facilitated other nuclease systems to achieve precise targeted integration. Moldt et al. (2008) employed a site-directed trans-acting Flp recombinase from IDLV transduction to integrate circular DNA produced by HIV-1 based LV transduction in the same cell. This approach could improve the integration of circular episomal DNA that is produced by the deficient integrase of the IDLV, or other HIV-1 based LV. In another study, Cornu and Carthomen delivered I-SceI nuclease and the repair template, *i.e.,* eGFP using IDLV with different mutations of IN into different cell lines (Cornu & Cathomen, 2007). Genotypic characterisation unfolds the integration of EGFP into the DSB site created by I-SceI. Transposase and transposable elements can also be utilized to deliver the desired sequence into the cell genome. Using IDLV, Vink et al. (2009) delivered an IR-flanked expression cassette for transposition that expressed Sleeping Beauty (SB) transposase from the generated episomal lentiviral DNA. Due to the exclusive integration mechanism of SB transposase and integration-deficiency of IDLV, the system avoided viral post-transcriptional regulator to improve vector titre and reduced random integration at active genes in cells. In a study where lentivirus-derived nanoparticles are used to deliver piggyback (PB) DNA transposase, the PB transposase is fused to the defective IN protein in C-terminal end of gag-pol and successfully integrate puromycin resistant gene into the cell genome *via* puromycin screening (Skipper et al., 2018). This study proves the possibility to add functional value to the defective integrase in IDLV.

### Immunization or vaccination

Immunization against a pathogenic infection or cancer disease can be initiated *via* vaccination of weakened infectious agent or part of the targets (Hussain, 2019; Levine & Sztein, 2004). The presentation of foreign agent to the body immune system is delivered by IDLV *via* the pseudotyping envelope membrane of the vector or the expression of the delivered transgenes. Early study attempted immunizing BALB/c mice with self-inactivating IDLV carrying codon-optimized HIV-1JR-FL gp120 sequence to induce an immune response against HIV-1 and generation of gp120 specific antibodies (Negri et al., 2007). In another study, a single inoculation of IDLV carrying gene encoding secreted form of the envelope of virulent strain of West Nile Virus (WNV) into mice induces robust B cells response and protection against a lethal dose of WNV and long-lasting immunity (Coutant et al., 2008). Retroviral or lentiviral vector pseudotyped with hepatitis C (HC) E1 and E2 glycoproteins, aka HC virus pseudoparticles (HCVpps), are introduced to the immune system to induce an immune response. Deng et al. (2013) has successfully immunized mice *via* inoculation of HCV E1/E2 pseudotyped IDLV bearing HCV NS3 gene. The presence of anti-E1, E2 and NS3 antibodies in the mice sera after vaccination shows neutralization of various HCVpp subtypes (1a, 1b, 2a, 3a and 5a).

Another application on influenza virus by introducing pseudotyped IDLV with virus hemagglutinin (HA) and nucleoprotein (NP) to CB6F1 mice stimulated production and maintenance of neutralizing antibodies level up to 18 weeks post-vaccination (Gallinaro et al., 2018). Apart from pseudotyping, the expression of antibodies transgene carried by IDLV also elicits immune responses towards the targeted pathogens. Michelini et al. (2020) vaccinated mice with engineered IDLV carrying genes encoding for VN04-2 monoclonal antibodies (mAbs) against influenza A virus (IAV). Expression of VN04-2 mAbs in the vaccinated mice provides some protection against H3N2 IAV, even though the mAbs are specific H5 HA. Recently, the global effort on COVID-19 vaccination has been an urgent concern. Yin et al. (2020) studied the immunization effectiveness of lentivirus-derived virus simulating particles (VSPs) decorated with SARS-COV-2 spike protein on the surface and carrying spike encoding mRNA in C57BL/6J mice. Elicitation of specific IgG towards spike protein was found in the vaccinated mice and a single injection is sufficient to induce immediate and potent immune responses against SARS-CoV-2. Recently, Ku et al. have actively studied the production of IDLV derived vaccines for current infectious diseases. For example, Ku et al. (2020) have designed a single dose IDLV-based ZIKA virus vaccine, carrying ZIKA viral genes for pre-membrane and envelope, demonstrating its immunizing effect on mouse models (A129 and BALB/c mice) as early as 7 days post immunization and long-lasting production of neutralizing antibodies for at least 6 months after immunization, without extra adjuvant formulation. In another study, Ku et al. (2021c) immunized mice and golden hamsters with vaccine derived from both integrative and non-integrative LV carrying spike glycoprotein gene displays strong vaccination efficiency and deleterious injury by eliciting an immune response in the respiratory tract. On the recent Covid-19 pandemic, Ku et al. (2021a) also attempted to create a new line of hACE2 transgenic mouse strain with high brain permissiveness to SARS-COV-2 replication (namely B6.K18-hACE2^IP-THV^) and tested the sterilizing protection in the lungs and brain of transgenic mice by intramuscular prime and intranasal boost of IDLV vaccine encoding spike glycoprotein of ancestral SARS-COV-2 and other variants. This study distinctively provided new insights of the protection of nervous system from the viral transmission through neuron invasion in the olfactory mucosa, addressing the prevention of neurological effects from infections that was less addressed previously.

Apart from pathogens, immunization against cancer using integrative LV has been implemented and shown to have comparable production of cancer specific immune cells to the commonly used adenoviral vector, as well as having more efficient expressions, and better quality of immune cells production due to little-to-no pre-existing antibodies towards lentiviral vector in human bodies (Ku et al., 2021b). Ku, Charneau & Majlessi (2021) had also previously reviewed in the context of vaccination using integrating LV. Nonetheless, applications of IDLV for cancer vaccination have also been studied actively. Morante et al. (2021) generated IDLV that carries ovalbumin (OVA) gene as a non-self-model antigen of lymphoma and melanoma, and TRP2 gene as a self-tumour associated antigen of melanoma and applied in the preclinical murine model. As a result of a single vaccination, tumour growth could be eradicated and/or controlled in lymphoma and melanoma expressing OVA within the studied period of 140 days. Also, long-lasting specific cytotoxic T cell response for months can also be activated by a single injection of IDLV carrying OVA as a model antigen, suggesting anti-tumour immunity (Cousin et al., 2019). This persistence is associated with the NF-kB signalling from the maturation of dendritic cells (DCs) and the production of inflammatory chemokines and cytokines.

Merck Sharp & Dohme Corp. has done a first-in-class, first-in-human clinical trial using a DC-tropic IDLV (codenamed LV305) to enable the expression of New York Esophageal Squamous Cell Carcinoma-1 (NY-ESO-1) cancer testis antigens in patients with sarcomas and other solid tumours (ClinicalTrials.gov Identifier: NCT02122861) (Somaiah et al., 2019). While the administered IDLV305 in outpatient cancer patients were tolerated well with only grade 1 or 2 treatment-related adverse effects, it also induced broad, tumour-specific, and durable anti-NY-ESO-1 immune response. The study indicates tumour regression over time in synovial sarcoma patient who achieved a near complete response under RECIST criteria. Upon this successful trial, subsequent ongoing clinical studies on combinations with G305, a cancer vaccine and anti-PD-L1 checkpoint inhibitor are executed (ClinicalTrials.gov Identifier: NCT02609984, NCT02387125).

### Cell reprogramming

IDLV could be used to reprogram cells to generate induced pluripotent stem cells (iPSCs). iPSCs are stem cells generated directly from mature somatic cells *via* introducing reprogramming factors (RF) (Robinton & Daley, 2012; Yamanaka, 2012). The earliest RFs discovered by Takahashi & Yamanaka (2006), *i.e.,* SOX2, OCT3/4, MYC2 and KLF4, aka Yamanaka’s factors, have been studied and improvised to achieve more efficient production of iPSC. While retroviral transduction was used by Yamanaka to reprogram the cells, Mali et al. (2008) has successfully reprogrammed human fibroblast to a pluripotent state that resembles human embryonic stem (hES) cells by IDLV transduction of the original Yamanaka’s factors with the addition of SV40 Tag. The reprogramming efficiency increased by 23–70-fold and the generated hESCs-like cells were able to differentiate into three embryonic germ layers in the embryoid body and teratoma formations. Fazlina attempted to introduce Yamanaka’s factors to U937 cells *via* LV and IDLV to induce pluripotency (Nordin, 2011). Although the expression level of the RF using IDLV is lower than LV due to lower viral vector production, mutations in IN enzyme did not prevent the expression of the RFs. While using LV, Papapetrou & Sadelain (2011) devised a different strategy to reprogram different types of cells using a single polycistronic lentiviral vector that expresses the Yamanaka’s factors but flanked by loxP sites between LTRs. Then, the expressed products are then excised by Cre recombinase delivered by IDLV transduction and converted into individual RFs that reprogram the cells into iPSCs in 12-14 weeks. Other than generating iPSCs, IDLV is also used to purify specific post-differentiated iPSCs. Yang et al. (2013) employed both LV and IDLV carrying GFP driven by liver-specific apolipoprotein A-II (APOA-II) promoter to sort and select human hepatic progenitor cells. The purified cells can express hepatoblast markers and further differentiate into more mature cells that secrete albumin. Other than iPSCs, cell programming of murine bone marrow-derived cells (BMDCs) to retinal pigment epithelium (RPE)-like cells is initiated *via Rpe65* and *Carlbp* expression, induced by IDLV expressed human RPE65 (Pay et al., 2018). For acute myeloid leukaemia, an immunotherapy based on reprogrammed monocytes with IDLV expressing GM-CSF, IFN- *α* and antigen was developed (Bialek-Waldmann et al., 2019). The reprogrammed monocytes self-differentiated to induced dendritic cells that migrate to lymph nodes and facilitate de novo T and B cells response regeneration.

### Cell death

Although IDLV is mainly studied for site-directed gene editing, transient expression gene therapy and immunization, there is also other application exploiting IDLV in the delivery and transduction of transgenes, thereby promoting their downstream processes, *i.e*., cell death. Vargas Jr, Klotman & Cara (2008) conditionally promoted cell death, by combining ganciclovir (GCV) treatment in SV40 trans-acting T antigen (Tag) and thymidine kinase (TK)-expressing cells. The transcriptionally active episomal vectors of TK suicide gene converted GCV to cytotoxic substance upon expression. When compared to IN-proficient LV transduction, IDLV transduced 293T cells presented similarly low survivability under the cytotoxicity effect induced by GCV treatment. Such principle could be applied for selective elimination of tumour cells during pro-drug treatment that is converted to toxic substance by an enzymatic function introduced to the transduced tumour cells.

## Limitations and prospects

IDLVs have improved safety characteristics compared to other integrating vectors encouraging clinical implementation, especially gene therapy, site-directed gene editing, cell reprogramming, immunization, and vaccination. Although IDLV has somehow eliminated the phenomena of transgene integration into the cell genomes, side effect like low expression of desired transgenes may still haunt the realization of clinical application. Efforts to reduce integrations are still appreciated while designing lentiviral vectors for gene therapies and other applications (Poletti & Mavilio, 2021). While the structure and the chemistry of the integrase enzyme have been described, modifications of the enzyme or the combination of these modifications would suggest even reduced integration (Engelman, 1999; Engelman, 2011; Maertens, Engelman & Cherepanov, 2021). These may include modifications that involve 3′ processing of transgenes, strand transfer activity and binding of LEDGF/p75 protein. A 12 base pairs deletion mutation of U3-LTR att site has also led to a greater-than-2-log reduction in vector integration and higher level of transgene expression (Shaw et al., 2017).

Another investigation of the extent of deletion in the transfer vector U3 region showed no influence in the integrase-mediated or -independent integration efficiency while removing such viral elements (Bona et al., 2021). Novel strategies to inactivate the integration reaction, such as new mutations affecting the involving elements are essential to further improve its safety of usage in clinical application, thus corroborating little to no side effects (Banasik & McCray, 2009). Regulation in the expression of transgene may also contribute to safety during post-transduction. For example, specific transcriptional or post-transcriptional controls, such as regulating microRNA or siRNA, could be designed to be co-transcribed with the transgenes. These regulating molecules may act on the transgenes directly or work together with other intrinsic mechanism in the transduced cells or other cells to restrict over-activity (Merlin & Follenzi, 2019). Like the use of short hairpin RNA (shRNA) delivered by LV to silence the BCL11A mRNA in sickle cell disease (Esrick et al., 2021), this feature can be implemented in the current IDLV setup with delayed or controlled transcription level to limit the transgenes expression. With similar aim, a photo-activating switch can also be utilized to turn-on the transduction mechanism by irradiating UVA rays on cells treated with LVs that are incorporated with photo-cleavable molecules within the vector’s envelope protein (Wang et al., 2019). In fact, the safety considerations for gene editing and gene therapy products have been discussed from FDA’s perspective (Reiser, 2017).

On the other hand, many current studies are limited to research settings and LVs are produced in laboratory scale and grade, which are often costly and contaminated with other substances. The concern of production and purification of large volume, high titre, current good manufacturing practice (cGMP)-grade lentiviral vectors would need to be addressed for real-world clinical application in human patients (Ausubel et al., 2007; Gandara, Affleck & Stoll, 2018; Merten et al., 2011; Milone & O’Doherty, 2018). An optimized purification protocol for lab scale LV production involving sequential chromatographic steps has been delineated to yield improved efficiency in *ex vivo* gene transfer, editing functions and decreased *ex vivo* and *in vivo* inflammatory response (Soldi et al., 2020). Another study described purification and concentration of LV in a pilot scale by employing Mustang Q chromatography, Tangential Flow Filtration and diafiltration (Tinch et al., 2019).

Apart from levelling up the production scale and grade, the preparation of patient condition or assisted-gene therapy is also essential to ensure effective treatment. Although effort using magnetic field *via* magnetofectins to increase LV cell transduction efficiency is only limited to *in vitro* application, it showed no overt cytotoxicity while maintaining cell integrity with reduced vector dose and incubation time (Orlando et al., 2010). The use of immunosuppressants, such as cyclosporine A (CsA) and Rapamycin (Rapa) has increased the transduction rate of LV or IDLV in hematopoietic stem and progenitor cells *via* action on the viral capsid (Petrillo et al., 2015). Petrillo et al. (2018) has attempted applying cyclosporine-derivatives, cyclosporine H (CsH), which is not immunosuppressive, on hematopoietic stem cells and it enhances IDLV-mediated gene transfer. The success behind is contributed by CsH abrogating the activity of interferon-induced transmembrane protein 3 (IFITM3). Such efforts should be considered to ensure the success rate of gene therapy and in the meantime lessen the side effects generated by the vector.

To further justify IDLV in clinical therapeutic implementation, its production and applications should be properly regulated. Although there are no specific regulatory guidelines to produce IDLV for clinical applications, the established guidelines from FDA and EMA on manufacturing lentiviral vectors (CHMP/BWP/2458/03) would be a reliable reference (2005). Meanwhile, White et al. (2017) has also summarized several extant regulatory guidance for advanced therapeutic medicinal products by FDA or EMA, specific issues on regulating gene therapies, stakeholders, and documentations that are involved during the clinical trials authorization of new lentiviral vector-mediated gene therapy. In general, safety testing during the preclinical trial of therapy should meet several goals, such as the proof-of-concept of the therapy, dose-response on efficacy, safe dosage, dose-escalation, drug distribution in the body, potential toxicity and its toxic effects, risks of adverse immune response, risks of specific population for inclusion/exclusion, persistence of gene expression, unintended aberrant localization or trafficking, risks of insertional mutagenesis, germline transfer, viral shedding, generation of replication-competent virus, sterility of preparation, and structural integrity of the genetic material. Reference to the success of regulated gene therapy medicine, such as Strimvelis™ for adenosine deaminase (ADA)-deficient severe combined immunodeficiency (SCID) and Zynteglo for beta thalassemia, would also assist considerations accounting for potential IDLV application in clinical studies (Aiuti, Roncarolo & Naldini, 2017; Schuessler-Lenz, Enzmann & Vamvakas, 2020)

## Conclusion

This review has first described the biology of IDLV, compared the differences between IDLV and other popular vectors, introduced some existing applications of IDLV, and enlisted some limitations and prospects of IDLV. IDLV has been introduced and appraised as a safer alternative to its carcinogenic integrating counterpart due to its defective integration process of transgene during cell transduction. The mutation introduced to the IN enzyme, or the binding sites has reduced the rate of insertional mutagenesis of the transduced cell genomes, producing stable episomal vector DNA and thus maintaining genomic stability and fitness. As these episomal vectors may be diluted in proliferating cells, the transient expression may be sustained in non-dividing cells. In addition, repercussions from using IDLV, such as the low efficiency of its transducibility has been addressed by strategies devised to boost its functionalities. Nonetheless, further effort or studies should be continued to overcome such issues in specific applications and elucidate the underlying reactions. While it is safer, pseudotyping and prudent transgene vector design of IDLV would provide flexibility and control over transduction of specific cells at desired conditions, making them an ideal choice of vector for delivery. Combined with other advanced technology such as nanotechnology and molecular editing mechanisms, the intrinsic characteristics of IDLV may be advantageous in inducing *de novo* expression, eliminating or repairing functions, or even encouraging new avenues apposite to health concerns.

## Additional Information and Declarations

### Data Availability

The following information was supplied regarding data availability: There is no raw data or code for this literature review.
